# The slime hand illusion: Nonproprioceptive ownership distortion specific to the skin region

**DOI:** 10.1177/20416695221137731

**Published:** 2022-11-15

**Authors:** Kenri Kodaka, Yutaro Sato, Kento Imai

**Affiliations:** 12963 Nagoya City University, Japan; 12963 Nagoya City University, Japan; 12963 Nagoya City University, Japan

**Keywords:** nonproprioceptive ownership distortion, mirror-visual feedback, slime hand illusion

## Abstract

The “slime hand illusion” is a simple and robust technique that uses mirror-visual feedback to produce a nonproprioceptive ownership distortion. The illusion can be easily evoked by the participant watching the experimenter pinching and pulling a chunk of slime in a mirror while the participant's hand, hidden behind the mirror, is similarly manipulated. This procedure produces a feeling of one of their fingers or the skin of their whole hand being stretched or deformed in a similar way to the visible slime. A public experiment found that more than 90% of participants reported a strong sense of skin or finger stretching. This report details a laboratory experiment performed to characterize the mechanisms behind the illusion more robustly. It reproduced this result and found that participants experienced a drift in their sense of skin location of approximately 30 cm on average, which is beyond the conventionally accepted range of proprioceptive drift.

Various techniques related to the rubber hand illusion (RHI; Botvinick & Cohen, 1998) paradigm have been shown to achieve distortion of hand ownership. In the RHI, a rubber hand is visibly touched simultaneously with the participant's hidden hand being physically touched. This creates an illusory sense of ownership of the rubber hand. The illusory effect is generally significantly reduced when the distance between the hands exceeds approximately 30 cm (Erro et al., 2020; Lloyd, 2007), corresponding to the boundary of the visuo-tactile peripersonal space surrounding the hand. This limitation arises essentially because the body image based on the proprioceptive signal resists being integrated into the vision-driven body image over such a large gap. Ownership distortion has frequently been found to involve proprioceptive drift toward the rubber hand, filling some or all of the gap between the two hands. The average proprioceptive drift is generally about 3–5 cm (Botvinick & Cohen, 1998; Durgin et al., 2007; Erro et al., 2020; Holmes et al., 2004; Rohde et al., 2011) but can reach almost 10 cm when the dummy hand is associated with the real hand through mirror-visual feedback (MVF; Tajima et al., 2015) or the illusion is created with the participant's eyes shut (Davies et al., 2013; Ehrsson et al., 2005; Kodaka & Ishihara, 2014). It is rare to see proprioceptive drift of over 10 cm.

Recently, we discovered a novel body-ownership illusion, the slime hand illusion (SHI; Sato et al., 2021) that seems to partially deviate from the conventional RHI paradigm in its effects. This illusion is based on the well-known procedure of MVF and can be seen in the video at https://youtu.be/w2K-VtuokBQ. A chunk of slime is placed in front of a mirror, the physical hand is placed behind the mirror, and the participant looks in the mirror so that they see the slime at the location of their own hand. Three types of illusory ownership distortion can be evoked: stretching of the skin, stretching of a finger, and hollowing out of the hand. To stretch the skin, the experimenter pinches the slime and pulls it upwards by typically 5–20 cm while performing the same movement on the hidden hand but remaining within the skin's physical constraints (stretching by ∼2 cm). To stretch a finger, the experimenter pinches the edge of the slime and pulls it away from the participant, typically by 5–50 cm, while only the tip of the participant's little finger is pulled in the same way. Note that the tactile operation performed on the hidden hand is similar to a former report (Byrne & Preston, 2019; Newport et al., 2015; Preston & Newport, 2011), concerning the illusory finger stretch, where the magnitude of the illusory finger stretch was at most twice the length of the real finger. To give the illusion of a hollowed-out hand, the experimenter bores a hole into the slime, while a nearly identical procedure is performed on the hidden hand. We have frequently found that participants report experiencing the illusion of stretched skin or a stretched finger to a distance matching the visual condition. In addition, more surprisingly, an almost identical effect occurs even when no slime is used and the hand operation is performed in midair in front of the mirror (the invisible SHI), similar to the invisible hand illusion (Guterstam et al., 2013), the invisible finger stretching illusion (Byrne & Preston, 2019), or the magnetic touch illusion (Guterstam et al., 2016). We hypothesized based on this empirical observation that the SHI has four unique characteristics that differ from those of the conventional RHI paradigm. The first is the extremely long deformation distance (more than 20 cm), the second is adaptability to an anomalous hand shape (e.g., the hollowed-out hand), the third is nonproprioceptive deformation (stretching of the skin), and the fourth is an extremely low degree of individual difference in the effect.

The effectiveness of the SHI and invisible SHI was partially demonstrated in a public experiment (*N* = 95) at the NTT InterCommunication Center (Shinjuku, Tokyo). More than 90% of participants rated the illusory skin or finger stretch at +2 or +3 on a seven-point Likert scale from −3 to +3 for the SHI, and more than 80% did so for the invisible SHI (Figure 1). Nearly 50% of participants (46/95) provided ratings in that range for the illusory hollowed hand, the first report of this specific body-image distortion as far as we know. Though the experiment was not performed under fully controlled conditions, the results strongly suggest that the SHI involves nonproprioceptive deformation, adaptability to an anomalous hand shape, and a marked lack of individual differences in the effect. We hypothesize that these unique characteristics are mainly driven by the illusion working on the part of the body-image specific to the skin. This is partially supported by the fact that participants did not report, orally or in questionnaire responses that the pinch-and-pull operation evoked upward or horizontal kinesthetic sensation of the entire hidden hand. It does not mean that the SHI does not involve the proprioceptive drift, because the proprioceptive drift does not generally involve kinesthetic sensation. The main idea behind our hypothesis is that the subjective skin-location drift in SHI contains two significant body-image distortions: proprioceptive drift and subjective skin deformation.

**Figure 1.**
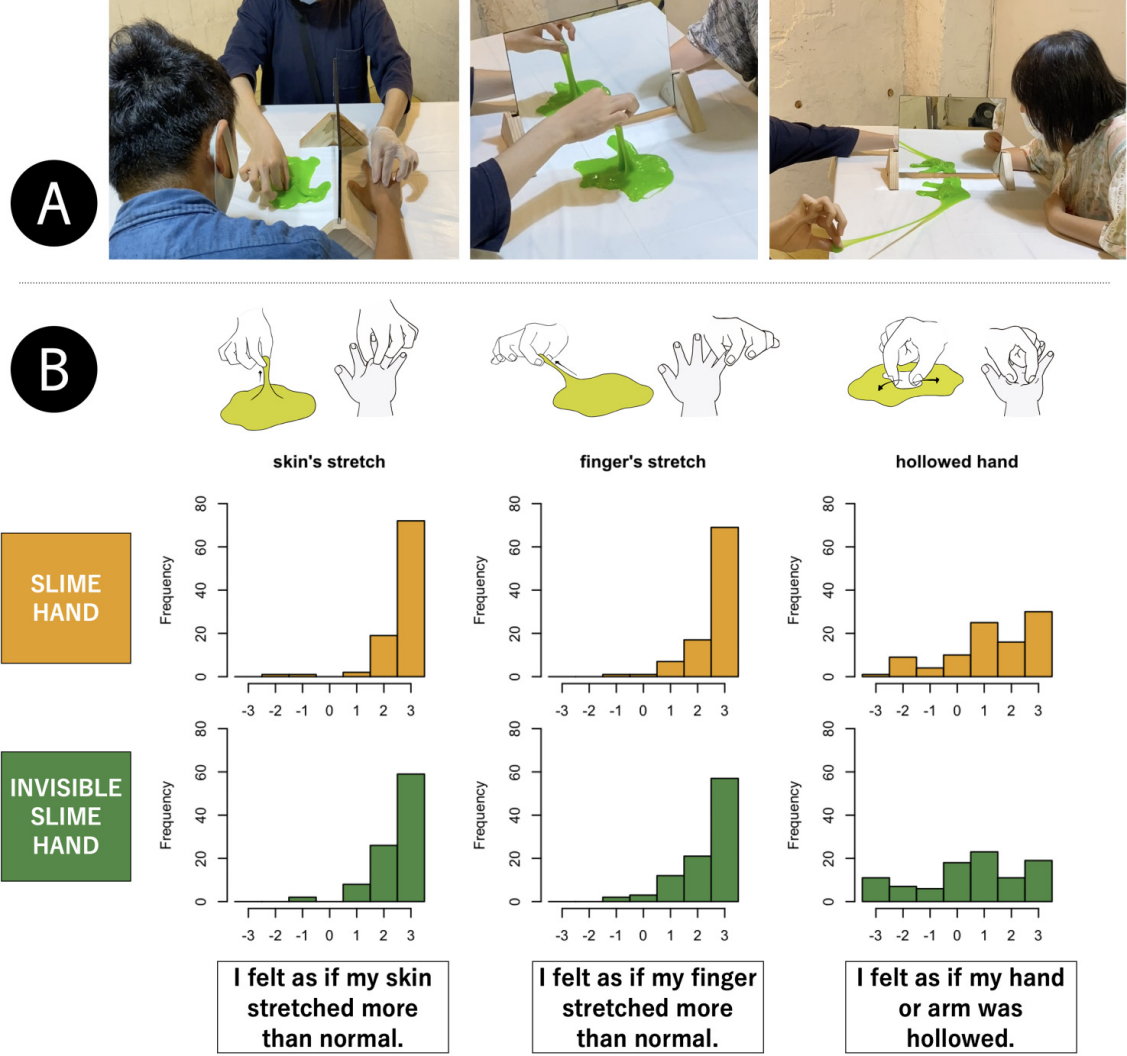
(A) Demonstration of the slime hand illusion in the laboratory, captured from https://youtu.be/w2K-VtuokBQ. (B) Histograms of illusion strength for each condition in the public experiment. In this experiment, nearly half of the participants experienced the SHI first, followed by the invisible SHI, and the remainder experienced the opposite order. For each condition, the participants experienced the three kinds of ownership distortion for approximately 3–5 min. After two trials, they were asked to provide subjective rating of the strength of the illusion on a 7-point Likert scale from −3 (*did not feel at all*), via 0 (*neither felt nor did not feel*), to +3 (*felt very strongly*).

To test some of these hypotheses more robustly, we performed a laboratory experiment using an MVF setup. The experiment had two main objectives. The first was to confirm the reproducibility of the SHI. The second was to decompose the slime-driven body-image distortion into proprioceptive drift and skin deformation and to evaluate the degree to which the SHI creates the illusion of skin deformation separately from proprioceptive drift. These purposes were achieved by creating two types of visuo-tactile correlations through manual operations on the slime and the hidden hand: the slime seen in the mirror was either partially pinched and pulled laterally with deformation (illusion condition) or laterally slid as a whole without deformation (control condition). Each of the two visual motions was temporally correlated with the same tactile operation on the hidden hand, where the lateral edge of the hand was pinched and pulled laterally without sliding the entire hand. The experiment was divided into two stages: agreement evaluation and subjective body-location evaluation. In the agreement evaluation, the participant was asked to rate five statements after seeing the deformed or undeformed slime operation for 60 s. In the subjective body-location evaluation, the participant was asked where the pinched skin or the nail of the little finger was felt to be after a 40-cm deformed or undeformed movement (UM) of the slime.

## Method

Twenty-three undergraduate students (6 males and 17 females; 23 right-handed) participated in the experiment. They were all recruited from the Faculty of Design and Architecture, Nagoya City University, and gave informed consent before participation. All participants received a book of tokens as compensation (1000 Yen). The protocol was approved by the ethics committee of Nagoya City University.

The initial setup of the experiment was as follows. A chunk of slime (colored green, 120 g) was placed on a color-striped sheet (30 × 57.5 cm) to the left of an upright mirror (30 × 45 cm), and the right hand of the participant (termed the hidden hand) was rested on the table to the right of the mirror, hidden from the participant's view behind the mirror and a blackout curtain. The slime was constrained to a circular shape by a 12.5-cm-diameter thin transparent-polypropylene cap (invisible to the participant). The outer edges of the slime and the hidden hand were each 15 cm from the mirror. The color-striped sheet was designed to allow the participant to give a rough estimate of the subjective location of a body part during the session. The sheet featured black, white, brown, blue, light blue, green, peach, orange, red, and yellow stripes from the mirror outward, all of which were 5 cm wide except for the black, which was 12.5 cm wide. The outer edge of the slime was therefore aligned with the middle of the white stripe.

**Figure 2.**
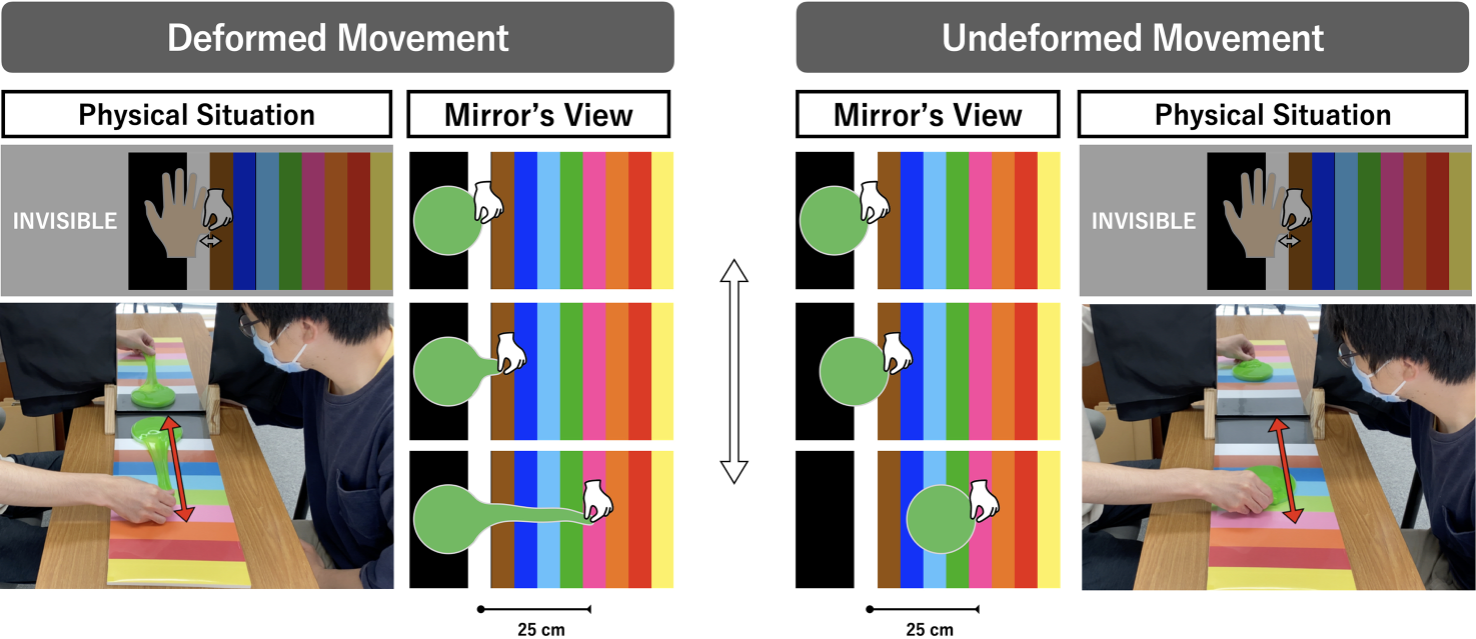
Photographs and schematic views of the agreement evaluation stage, where they saw the slime operated on during two types of visuo-tactile correlations (deformed and undeformed movement) with the hidden hand.

Two kinds of visuo-tactile correlations were created between the slime and the hidden hand through the experimenter performing manual operations with their two hands (Figure 2). In both conditions, the lateral edge of the hidden hand was pinched and pulled laterally without sliding the hand and with tactile pressure consistent with the observed movement of the slime. In UM, the outer edge of the slime was pinched, and the slime was slid laterally as a whole toward a specific location. The slime was then returned to its original position. Note that there was no deformation of the slime in UM because the movement was achieved by sliding the transparent support under the slime. In deformed movement (DM), the experimenter pinched and laterally pulled the outer edge of the slime firmly to stretch it toward a specific location without tearing it. The stretched slime was then quickly returned to its original starting point without touching the desk and reformed into the original circular shape. This partial deformation was enabled by fixing the transparent support firmly onto the color sheet.

**Figure 3.**
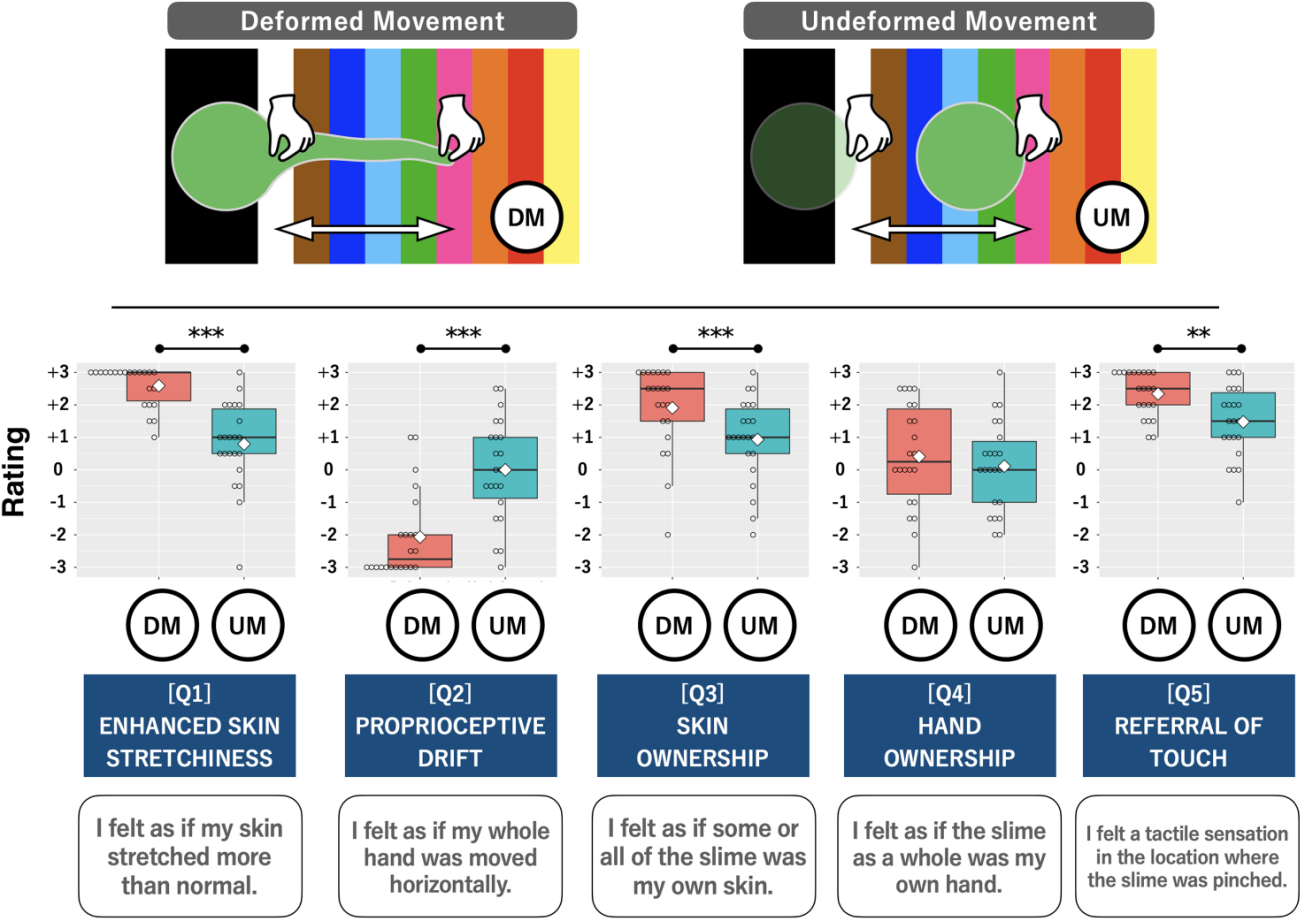
Results of the agreement evaluation stage, where the participant was asked to rate five statements after a 1-min session in which they saw the slime operated on during two types of visuo-tactile correlations (deformed and undeformed movement) with the hidden hand. Bold lines indicate the medians; white diamonds indicate the average; upper and lower limits of the box plot indicate the 75 and 25 percentiles. Asterisks indicate that significant differences between two movements in a paired t-test (∗*p* < .05, ∗∗*p* < .01, ***p < .001).

The experiment was divided into two stages: agreement evaluation and subjective body-location evaluation. In the agreement evaluation, UM or DM was repeatedly performed for 60 s with a maximum displacement of the slime from its original position of 25 cm (the middle of the pink stripe). After each session, the participant was asked to rate five statements as shown in Figure 3 on a 7-point Likert scale ranging from −3 (*did not feel at all*), via 0 (*neither felt nor did not feel*), to + 3 (*felt very strongly*). The agreement evaluation comprised two UM and two DM sessions, with UM and DM performed in turn and with the initial condition counterbalanced. In the subjective body-location evaluation, the slime was moved a fixed distance of 40 cm (to the middle of the yellow stripe) via UM or DM, and the participant was asked to hold their attention exclusively on one of two specific parts of the hidden hand: the nail of the little finger (NL) or the skin being pinched (SP). Once before the movement (preillusion position) and again shortly after the one-way lateral movement (postillusion position), participants indicated orally which color was behind their subjective NL or SP, while seeing the striped sheet viewed in the mirror. For the postillusion measurement of SP only, the participant was asked to indicate which color corresponded to the most extended position reached during the one-way movement because we found that, for a few individuals participating in a preliminary experiment, the subjective position of the skin instantly reverted to normal once their attention was taken off the slime. The color indicated was then converted to a numerical measure as follows: −5, 0, +5, +10, +15, +20, +25, +30, +35, +40 cm for black, white, brown, blue, light blue, green, peach, orange, red, and yellow, respectively. Finally, the drift of the participant's sense of location for NL (proprioceptive drift) and SP (subjective skin-location drift) was calculated by subtracting the preillusion position from the postillusion position. The subjective body-location evaluation stage comprised four blocks mixing the two factors of visual operation (UM, DM) and attentive target (NL, SP). The procedure was repeated four times in a row during a single block. The order of the four conditions was counterbalanced.

## Results

Data for one participant were excluded because of physical difficulty in pinching the skin at the edge of their hand. Data for the remaining 22 participants were analyzed as follows. Statistical significance was evaluated using an alpha value of 0.05.

### Agreement Evaluation

The effects of UM and DM on the five agreement evaluations were compared using paired t-tests. The rating distributions are shown in Figure 3. DM received significantly higher participant agreement ratings for a sensation of enhanced skin stretchiness (Q1: *t*(21) = 7.01, *p* < .001, Cohen's *d* = 1.74), feeling ownership of the slime as their own skin (Q3: *t*(21) = 4.18, *p* < .001, Cohen's *d* = 0.75), and tactile sensations being referred toward the slime (Q5: *t*(21) = 3.38, *p* < .01, Cohen's *d* = 0.93). Feeling proprioceptive drift was the only sensation for which DM received significantly lower ratings than UM (Q2: *t*(21) = 6.28, *p* < .001, Cohen's *d* = 1.42). Ratings of feeling ownership of the slime as their own hand were not significantly different between UM and DM (Q4: *t*(21) = 0.99, *p* = .33, Cohen's *d* = 0.20); it was rated at about zero (*neither felt nor did not feel*) for both conditions.

### Subjective Body-Location Evaluation

**Figure 4.**
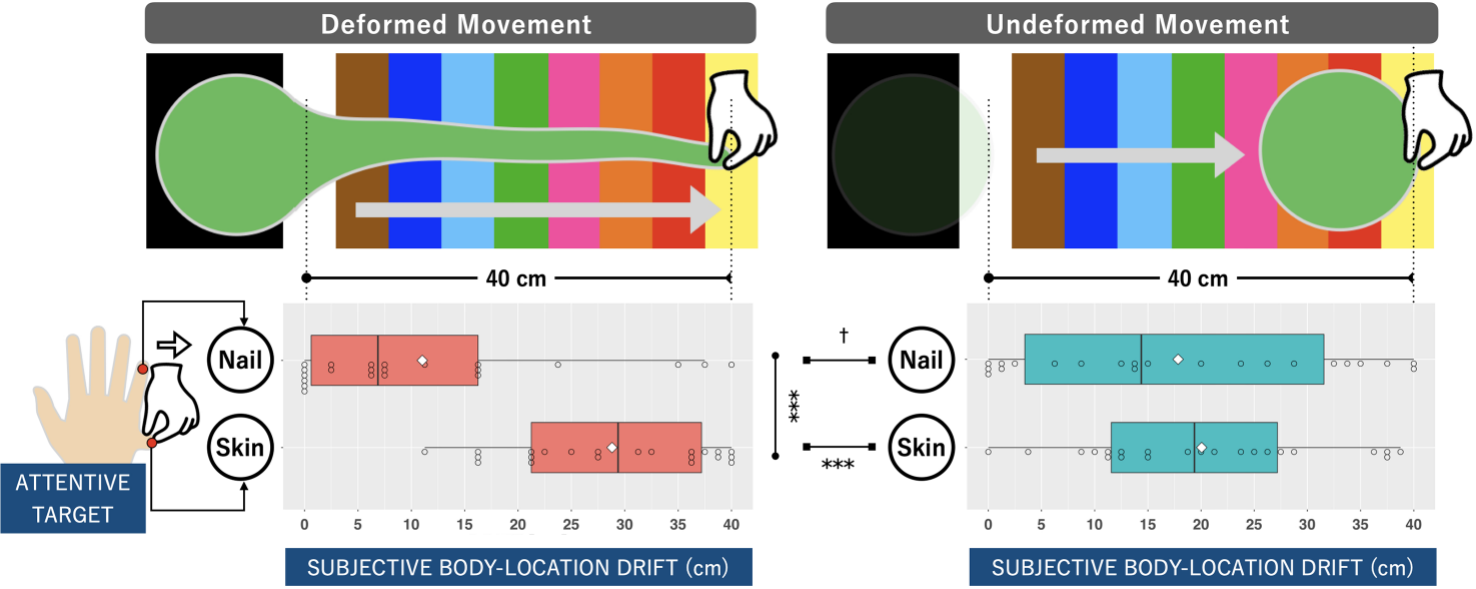
Results for the subjective body-location evaluation stage, where the participant was asked to evaluate their sense of the location of the hidden hand (nail or skin) prior to and after 40-cm lateral deformed/undeformed movement of the slime. The bar graphs represent the distribution of the average subjective drift of nail or skin location, which was calculated by subtracting the pre-illusion position from the post-illusion position for each participant. Bold lines indicate the medinas; white diamonds indicate the average; upper and lower limits of the box plot indicate the 75 and 25 percentiles. Asterisks indicate significant differences between two conditions found in simple main effect analysis (†*p* < .1, ∗*p* < .05, ∗∗*p* < .01, ***p < .001).

Drift in subjective body location (postillusion position minus preillusion position) was analyzed through two-way analysis of variance with the within-subjects factors of visual operation and attentive target. The comparative distributions of location estimations are shown in Figure 4. The analysis revealed a significant main effect of attentive target (*F*(1, 21) = 33.8, *p* < .001, $\eta_{n}^{2}$ = 0.62) and a significant interaction (*F*(1, 21) = 26.0, *p* < .001, $\eta_{n}^{2}$ = 0.55), both of which were large effects. In addition, follow-up analyses of the interaction effect showed that DM produced a medium-large marginally significantly drop in the drift of the sense of location for NL (proprioceptive drift) versus UM (*F*(1, 21) = 4.17, *p* = .054, $\eta_{n}^{2}$ = 0.17) but a significantly larger drift of sense of location for SP (subjective skin-location drift) than UM (*F*(1, 21) = 14.7, *p* < .001, $\eta_{n}^{2}$ = 0.41). The simple main effect of attentive target was found to be significant only for DM, where subjective skin-location drift was significantly larger than proprioceptive drift (*F*(1, 21) = 42.2, *p* < .001, $\eta_{n}^{2}$ = 0.67)). There was no significant difference between the magnitudes of the two drifts for UM (*F*(1, 21) = 1.59, *p* = .22, $\eta_{n}^{2}$ = 0.071)).

## Discussion

In this experiment, the DM illusion condition was designed to represent the original SHI. However, DM consisted only of simple back-and-forth deformation of the slime, which fell well short of the ‘full course’ of SHI seen in the video. Despite this limitation, 19 out of 22 participants rated the statement that their skin seemed anomalously stretchy (Q1) at +2 or more, on average, reproducing the finding that the SHI consistently yields the illusion of stretched skin. Importantly, while the geometrically circular slime was not felt to embody the whole hand of the participant, regardless of condition, as demonstrated in the poor ratings of Q4, part of the slime was felt to strongly embody the skin of the participant in the DM condition (a mean rating of 2.53 for Q3). Thus, skin ownership was found to be transferred to the external object even without the assistance of whole-hand ownership. This finding is compatible with the reported subjective experience of proprioception. A sense of proprioceptive drift was strongly denied in DM, where 17 out of 22 participants rated ‘I felt as if my whole hand was moved horizontally’ (Q2) at −2 or less, on average. This means the participants exclusively experienced skin deformation during the SHI; whole-hand movement was not the focus of their attention. Thus, the results for agreement evaluation strongly support our hypothesis that the SHI works specifically on skin perception.

This idea is also supported by the results from the subjective body-location evaluation. Proprioceptive drift was estimated by measuring the subjective displacement of the NL, which was on average about 10 cm in DM and 20 cm in UM. These distances are somewhat larger than in conventional reports (Botvinick & Cohen, 1998; Durgin et al., 2007; Erro et al., 2020; Holmes et al., 2004; Rohde et al., 2011; Tajima et al., 2015). We speculate this is partially because, in this experiment, the felt position was measured with the participant's eyes directed to the mirror's view containing the visual appearance of the slime, whereas the measurement of the proprioception before/after the RHI has been typically exhibited with the participant's eyes closed. All the same, this vision-driven proprioceptive drift both in UM and DM still lies within the distance between a real hand and a dummy hand that allows significant ownership distortion. On the other hand, the subjective skin-location drift in DM was estimated to be around 30 cm. Though it is not fair to directly compare this value of 30 cm with the conventional measurement of the proprioceptive drift yielded by the RHI (typically around 5 cm) because of the measurement difference, it is nevertheless true that this is far beyond the conventionally accepted range of proprioceptive drift. Thus, in keeping with the subjective impressions expressed in the agreement evaluation, subjective skin deformation can be interpreted to be the main component of the subjective body-location drift in DM. More importantly, 9 out of 22 participants estimated the magnitude of the subjective skin-location drift at more than 35 cm in DM, even though the actual length of slime deformation was 40 cm. This suggests that further subjective skin deformation may be possible. Thus, the experimental result strongly supports our hypothesis that the slime hand can be deformed beyond the peripersonal space specific to the hand.

Even though exactly the same tactile sensation was given to the hidden hand in DM and UM, the SHI was much less marked in UM. As high agreement evaluations were given for the invisible SHI in the public experiment (rated at 2.47 ± 0.82), the decline of the illusion can arguably be attributed purely to the visual expression of the slime specific to UM: undeformed visual translation is not consistent with the illusory skin deformation. This means that an appropriate visuo-tactile correlation (such as pinching and pulling the slime) maintains or magnifies the illusory effect of the invisible SHI but an inappropriate correlation (such as sliding the slime without deformation) significantly degrades the effect. It is especially notable that this rule regarding correlation is independent of the anatomical congruency that is an indispensable requirement of the RHI, as the appearance of the slime was different from the visual form of the hand but consistently evoked an SHI. Thus, it will be interesting to further explore the potential of the SHI by assessing which kinds of visual congruency affect it.

In summary, the laboratory experiment detailed here clearly reproduced the illusion of stretched skin previously reported in the SHI. Analysis indicated that skin ownership was transferred to a part of the slime without distorting whole-hand ownership. In addition, sense of skin-location drifted by about 30 cm on average, driven mainly by perceived skin deformation, not proprioceptive drift. We believe this to be the first comprehensive report of a body-ownership illusion specific to the skin region. It is an important starting point for exploring the mechanisms involved in the skin-ownership illusion, which is essentially free from the spatial limitation classically seen in proprioceptive distortion.
